# Sex chromosome complement regulates expression of mood-related genes

**DOI:** 10.1186/2042-6410-4-20

**Published:** 2013-11-07

**Authors:** Marianne L Seney, Kokomma I Ekong, Ying Ding, George C Tseng, Etienne Sibille

**Affiliations:** 1 Department of Psychiatry, University of Pittsburgh, Pittsburgh, PA 15213, USA; 2 Translational Neuroscience Program, University of Pittsburgh, Pittsburgh, PA 15213, USA; 3 Department of Biostatistics, University of Pittsburgh, Pittsburgh, PA 15261, USA; 4 Department of Computational and Systems Biology, University of Pittsburgh, Pittsburgh, PA 15260, USA; 5 Department of Human Genetics, University of Pittsburgh, Pittsburgh, PA 15261, USA; 6 Center For Neuroscience, University of Pittsburgh, Pittsburgh, PA 15261, USA

**Keywords:** GABA, Serotonin, Dopamine, Four Core Genotypes mice, Anxiety, Depression

## Abstract

**Background:**

Studies on major depressive and anxiety disorders suggest dysfunctions in brain corticolimbic circuits, including altered gamma-aminobutyric acid (GABA) and modulatory (serotonin and dopamine) neurotransmission. Interestingly, sexual dimorphisms in GABA, serotonin, and dopamine systems are also reported. Understanding the mechanisms behind these sexual dimorphisms may help unravel the biological bases of the heightened female vulnerability to mood disorders. Here, we investigate the contribution of sex-related factors (sex chromosome complement, developmental gonadal sex, or adult circulating hormones) to frontal cortex expression of selected GABA-, serotonin-, and dopamine-related genes.

**Methods:**

As gonadal sex is determined by sex chromosome complement, the role of sex chromosomes cannot be investigated individually in humans. Therefore, we used the Four Core Genotypes (FCG) mouse model, in which sex chromosome complement and gonadal sex are artificially decoupled, to examine the expression of 13 GABA-related genes, 6 serotonin- and dopamine-related genes, and 8 associated signal transduction genes under chronic stress conditions. Results were analyzed by three-way ANOVA (sex chromosome complement × gonadal sex × circulating testosterone). A global perspective of gene expression changes was provided by heatmap representation and gene co-expression networks to identify patterns of transcriptional activities related to each main factor.

**Results:**

We show that under chronic stress conditions, sex chromosome complement influenced GABA/serotonin/dopamine-related gene expression in the frontal cortex, with XY mice consistently having lower gene expression compared to XX mice. Gonadal sex and circulating testosterone exhibited less pronounced, more complex, and variable control over gene expression. Across factors, male conditions were associated with a tightly co-expressed set of signal transduction genes.

**Conclusions:**

Under chronic stress conditions, sex-related factors differentially influence expression of genes linked to mood regulation in the frontal cortex. The main factor influencing expression of GABA-, serotonin-, and dopamine-related genes was sex chromosome complement, with an unexpected pro-disease effect in XY mice relative to XX mice. This effect was partially opposed by gonadal sex and circulating testosterone, although all three factors influenced signal transduction pathways in males. Since GABA, serotonin, and dopamine changes are also observed in other psychiatric and neurodegenerative disorders, these findings have broader implications for the understanding of sexual dimorphism in adult psychopathology.

## Background

Major depressive disorder (MDD) is a common, chronic illness characterized by low mood, sadness, or irritability along with psychophysiological changes, ultimately interfering with family relations and normal daily activities [1]. Affecting approximately 14.8 million adults in the USA, MDD is twice as common in women as in men, with the lifetime incidence of MDD being 20% in women and 12% in men [1]. In addition to sex differences in prevalence and incidence of MDD, women have a higher morbidity risk than men [2]. Notably, community-based epidemiologic studies also report increased female prevalence of MDD [3]. Thus, these studies observe sex differences in MDD after controlling for the potential confounding variable of seeking treatment, suggesting underlying predisposing biological factors in females.

### GABA dysfunction in MDD

Studies suggest dysfunction of corticolimbic circuits and dysregulation in gamma-aminobutyric acid (GABA) neurotransmission in MDD. Magnetic resonance spectroscopy [4-6] and molecular [7,8] studies have directly reported decreased GABA content in MDD or suggested reduced GABA-mediated inhibition (reviewed in [9]). Our group recently reported reduced markers of dendritic-targeting GABA interneurons in the amygdala [8], subgenual anterior cingulate cortex (sgACC) [10,11], and dorsolateral prefrontal cortex (dlPFC) [12] of MDD patients, specifically affecting somatostatin (SST), the most common marker of dendritic-targeting interneurons. Interestingly, these findings were more robust in females with depression [10,11,13]. These human findings are supported by causal studies in mice, where mild reduction in GABA signaling is sufficient to induce depressive-like behaviors [14], together supporting a GABA deficit hypothesis of MDD [9]. We recently examined expression of *Sst*, and of the GABA-synthesizing enzymes glutamate decarboxylase 67 (*Gad67*) and *Gad65*, in the frontal cortex of Four Core Genotypes (FCG) mice in order to independently examine the contribution of developmental gonadal sex (organizational), adult circulating hormones (activational), and X/Y sex chromosome complement on expression of those genes under rodent conditions that are homologous to a human depressed state, i.e., after exposure to unpredictable chronic mild stress. Contrary to our prediction based on increased MDD prevalence in women, regardless of gonadal and adult hormonal status, FCG XY mice (i.e., genetic males that lack the sex-determining gene *Sry* on the Y chromosome; see 'Methods’) had lower expression of these three genes compared to XX mice, with concomitant elevated anxiety-like behavior; interestingly, adult testosterone treatment decreased anxiety-like behaviors, but did not affect *Sst*, *Gad67*, or *Gad65* gene expression [13]. Together, these studies in humans and mice support a general hypothesis of GABA dysfunction in MDD and point to sex chromosome complement as a potential modulator.

### Serotonin and dopamine in MDD

Along with evidence for dysfunction in fast-acting GABA neurotransmission in MDD, evidence also suggests problems with slow-acting neuromodulatory systems (e.g., serotonin and dopamine). Evidence for involvement of serotonin in MDD includes decreased tryptophan [15] and decreased serotonin metabolites [16] in the cerebrospinal fluid (CSF) of depressed patients and altered markers of transmission in the frontal cortex and of midbrain factors involved in serotonin production, suggesting ineffective subcortical-cortical serotonergic transmission [17]. Studies in rodents have extensively probed the link between the serotonin system and anxiety/depressive-like behaviors [18,19], and sex differences in the serotonin system have been reported in humans and rodents (e.g., [20-22]). Imaging and *postmortem* studies of the dopamine system in MDD suggest changes in receptor levels but are overall more equivocal [23-25]. Recent optogenetic studies suggest a causal link between ventral tegmental area (VTA) dopaminergic neuron firing and anhedonia-like behavior in mice [26]. Imaging studies in humans report dopamine-related sexual dimorphism in the frontal cortex of control subjects, with increased dopamine D_2_-like receptors in women compared to men [27]. Sex differences in dopamine have been extensively studied in rodents and report, for instance, lower dopamine levels and higher turnover in the frontal cortex of female compared to male rats [28].

### Biological sex factors

Taking into consideration potential hormonal mechanisms underlying observed sexual dimorphism, circulating gonadal hormones have acute, transient effects throughout life (activational hormone effects), while exposure to gonadal hormones during critical periods of development can cause permanent sex differences (organizational hormone effects). The organizational actions of gonadal hormones have long been known to cause sex differences in behavior and brain structure, although the majority of studies involve sex behavior and regions controlling sex behavior in rodents (e.g., [29,30]). Evidence from human studies suggests an association between low testosterone levels and anxiety and depression susceptibility, suggesting activational effects of testosterone on mood. For instance, a prospective study following men over 50 with low or normal levels of testosterone found that men with low testosterone had increased risk of developing depression [31]. Another study found lower testosterone levels in depressed elderly men compared to non-depressed controls [32]. Interestingly, evidence also suggests an association of testosterone levels with anxiety and depression in women and that SSRIs may increase testosterone levels in both men and women [33]. An additional mechanism underlying sexual dimorphism is sex chromosome complement; Y chromosome gene or X chromosome gene dosage could play a role in sexual dimorphism (reviewed in [34]). Sex chromosome complement has a predominant role during development in determining gonadal sex, but contributions of X/Y genes are ongoing throughout life. It is impossible to separate the role of sex chromosomes from gonadal sex in traditional wild-type mice, regardless of hormone manipulation. However, genetic manipulation was used to engineer the FCG mice, in which the testes-determining gene *Sry* was placed on an autosome after spontaneous deletion from the Y chromosome, thus dissociating sex chromosome complement from gonadal sex. In addition to our studies in the FCG mice detailed above, other studies in FCG mice have revealed sex chromosome influences on sexual differentiation of aggressive, parental, and social behaviors [35-37] and vasopressin innervation in the lateral septum [38].

Evidence suggests both dysregulation in human mood-related disorders affecting the GABA, serotonin, and dopamine systems in MDD and sexual dimorphism in these systems across species; however, the underlying causes for these sex differences remain unclear. Here, we used the FCG mice to probe frontal cortex expression of selected genes in the GABA, serotonin, and dopamine systems for evidence of sexual dimorphism and to investigate the putative sex-related origins of observed sex differences, with the goal of gaining insight into the biological basis of the sexual dimorphism of MDD and anxiety disorders. Since we are interested in identifying genes that may vary by sex in the context of mood disorders, we investigated FCG mice exposed to unpredictable chronic mild stress (UCMS). This model replicates the role of stress in eliciting physiological and behavioral changes that are homologous to a depressive-like syndrome in mice and respects the time frame of onset and efficacy of antidepressant treatment [39]. Based on the female prevalence of these mood disorders, we predicted that gene expression would be affected in pro-disease directions in the 'female’ phenotypes (XX mice, gonadal females, and mice not treated with testosterone in adulthood).

## Methods

### Mice

FCG mice were used in these studies (Jackson Laboratories, Bar Harbor, ME, USA; B6.Cg-Tg(Sry)2Ei Srydl1Rlb/ArnoJ). By crossing a C57BL/6 J female with an XY^-^*Sry* male (Y^-^ denotes absence of *Sry* on the Y chromosome; *Sry* denotes presence of the autosomal *Sry* transgene), four groups of mice are generated: XX males (XX*Sry*), XX females (XX), XY^-^ males (XY^-^*Sry*), and XY^-^ females (XY^-^). The mice were maintained under standard conditions of 12-h light and dark cycles (22°C ± 1°C, food and water *ad libitum*), in accordance with the University of Pittsburgh Institutional Animal Care and Use Committee. The numbers of mice investigated per group are indicated at the base of the bars in the figures.

These mice were used in a previous study investigating the effects of UCMS exposure on anxiety- and depressive-like behaviors in FCG mice [13]. All mice used here for gene expression analyses were sacrificed after chronic stress exposure. FCG mice were bilaterally gonadectomized at 15 weeks of age to remove endogenous sources of gonadal hormones and implanted with either a testosterone-filled (5-mm crystalline testosterone; 1.57-mm ID × 2.41-mm OD) or similarly sized blank SILASTIC capsule (Dow Corning Corp., Midland, MI, USA). This size testosterone capsule yields circulating testosterone levels at or slightly above normal male levels (N. Ghahremani, personal communication). At the time of sacrifice, we collected trunk blood for testosterone assay to confirm the efficacy of our adult hormone manipulation. Serum samples were sent to the University of Virginia Center for Research in Reproduction Ligand Assay and Analysis Core (supported by the Eunice Kennedy Shriver NICHD/HIH (SCCPIR) Grant U54-HD28934), and a radio-immuno assay (RIA) was used to determine testosterone concentration. A three-way analysis of variance (ANOVA) revealed that serum testosterone levels did not differ by sex chromosome complement (XX, 1,026.2 ± 405.2; XY^-^, 886.2 ± 647.2; *p* > 0.75) or gonadal sex (gonadal females, 471.0 ± 238.9; gonadal males, 1,453.6 ± 690.6; *p* > 0.15), but did differ by hormone manipulation (blank-treated, 225.8 ± 140.8; testosterone-treated, 1648.9 ± 687.1; *p* < 0.05).

### Unpredictable chronic mild stress

UCMS is a behavioral paradigm that robustly increases behavioral emotionality, thus presenting some homologous features associated with human depression (although not 'modeling’ this complex human disorder, but providing critical information about the system under chronic stress conditions) [40]. The UCMS protocol consisted of a 7-week period during which group-housed mice were exposed to a randomized schedule of environmental stressors approximately one to two times per day, 7 days a week, as applied in our lab [39,41,42]. Disturbances included forced bath (approximately 2 cm of water for 15 min), aversive smell (1 h of exposure to bobcat urine), light cycle reversal or disruption, social stress (rotate mice into previously occupied cages), tilted cage (45° tilt), mild restraint (50-ml conical tube with air hole for 15 min), bedding change (replace soiled bedding with clean bedding), wet bedding, and no bedding. Weekly assessment of weight and fur was performed to track progression of the UCMS syndrome (as in [39]). After 7 weeks of UCMS exposure, mice were run through a series of behavior tests to assess anxiety/depressive-like behavior: elevated plus maze, open field, and sucrose preference [13].

### Frontal cortex dissection

At the time of sacrifice, brains from FCG mice were flash frozen on dry ice (after 7 weeks of UCMS and while still being exposed to stressors) and stored at -80°C. Rostro-caudal sections (160 μm thick) were obtained using a cryostat, and a 1-mm-bore tissue punch was used to isolate the bilateral frontal cortex (cingulate cortex and prelimbic cortex; between bregma +2.34 and +0.50 mm; [43]).

### Gene selection

#### GABA-related genes

We examined expression of several genes associated with cortical GABA microcircuitry: (1) markers of dendritic-targeting interneurons (neuropeptide Y (*Npy*), cortistatin (*Cst*), vasoactive intestinal peptide (*Vip*)), (2) markers of perisomatic-targeting GABA neurons (parvalbumin (*Pv*), cholecystokinin (*Cck*)) and other interneuron-targeting interneurons (calretinin (*Cr*)), (3) other GABA-related genes (GABA A receptor subunits (*Gabra1*, *Gabra2*, *Gabra5*) and the GABA transporter (*Gat1*)), and (4) two brain-derived neurotrophic factor (BDNF)-related genes (*Bdnf* and its receptor, *Trkb*), based on previous results indicating BDNF control of GABA-related gene expression [8,11].

#### Serotonin- and dopamine-related genes

Expression of serotonin- and dopamine-related genes was examined in the Allen Brain Atlas. Genes with high frontal cortex expression were selected for quantitative polymerase chain reaction (qPCR) analysis. We examined expression of (1) serotonin and dopamine receptors (*Drd1a*, *Htr1a*, *Htr2a*, *Htr2c*), (2) associated signal transduction pathway components (adenylate cyclase 1 (*Adcy1*), *Adcy2*, *Adcy5*, *Adcy7*, cyclin-dependent kinase 5 (*Cdk5*), *Akt1*, *Akt2*, *Akt3*), and (3) related genes with prior evidence for sex differences (amyloid precursor protein (*App*) prodynorphin (*Pdny*) [44]).

### Real-time quantitative PCR

Total RNA was extracted from tissue punches using the Allprep® DNA/RNA Micro Kit (Qiagen, Valencia, CA, USA) and assessed by chromatography (Agilent Bioanalyzer, Santa Clara, CA, USA). RIN was 8.35 ± 0.04 (mean ± standard error of the mean (SEM)), indicating excel-lent RNA quality. Total RNA (100 ng) was reverse-transcribed into cDNA using QScript cDNA Supermix (oligo(dT) and random primers, Quanta Biosciences, Gaithersburg, MD, USA). Small PCR products (70–100 bp) were amplified on a Mastercycler® ep Realplex2 qPCR machine (Eppendorf, Hamburg, Germany) using universal PCR conditions (65°C to 59°C touch-down and 40 cycles (10 s at 95°C, 10 s at 59°C, and 10 s at 72°C)). cDNA was amplified in 15-μl reactions (0.1 × SYBR Green, 3 mM MgCl_2_, 200 nM dNTPs, 200 nM primers, 0.25 unit Platinum Taq DNA polymerase (Invitrogen, Carlsbad, CA, USA)). Primer-dimers were assessed by amplifying primers without cDNA. Primers were retained if they produced no primer-dimers or non-specific signal only after 35 cycles. Samples were run in quadruplicate and results were calculated as the geometric mean of relative intensities compared to two internal controls (actin and glyceraldehyde-3-phosphate dehydrogenase; these internal controls were not influenced by sex-related factors in our dataset). Results are expressed as arbitrary signal (2^-dCT^ × 10,000) [45].

### Statistical analysis of gene expression results

We used a full three-way ANOVA (sex chromosome complement × developmental gonadal sex × circulating hormone with all three first-order interaction and one second-order interaction terms) to compare groups for gene expression. This same approach to analyzing effects of sex-related factors has been previously used for FCG mice (e.g., [46-48]). Developmental gonadal sex effects were analyzed by comparing mice with ovaries to those with testes; since all mice were gonadectomized approximately 10 weeks prior to sacrifice, any gene expression differences observed due to gonads are considered to be organizational effects (permanent changes due to hormone exposure during a critical developmental period). Activational effects of testosterone were examined by comparing mice gonadectomized in adulthood and implanted with testosterone-filled capsules to those gonadectomized and implanted with blank capsules. If the three-way ANOVA was significant for any main effect or interaction, we performed planned contrasts using Tukey's *post hoc* test. Data are expressed as mean ± SEM, statistical significance was set at *p* < 0.05, and trend level was set at *p* < 0.1. To test for potential type I errors, we controlled the false discovery rate (set to 5%) using the Benjamini-Hochberg method [49]; the number of genes in each category was used as the total number of tests (13 for GABA-related genes, 14 for serotonin/dopamine-related genes). Note that the goal of this study was not to examine the effect of chronic stress on gene expression, but rather to investigate the effect of sex-related factors on gene expression under chronic stress conditions.

### Correlation between gene expression and anxiety-like behavior

Using the Pearson correlation, we compared expression of each gene examined here as well as for *Sst*, *Gad67*, and *Gad65* (reported in [13]) to anxiety-like behavior in the same chronically stressed mice (behavior reported separately in [13]). In order to reduce the complexity of this correlational analysis, we combined anxiety-like behavior measures from the elevated plus maze (time open and percent crosses into open arms) and open field (time center, percent distance center) into anxiety-like emotionality *Z*-scores (see detailed methods for behavioral Z-scoring in [50]). *Z*-scores calculate how many standard deviations (*σ*) an observation (*X*) is abovp (*μ*):

$$z=\frac{X-\mu }{\sigma }.$$

First, we calculated *Z*-scores for each behavior test measure (e.g., Z_EPM_TimeOpen or Z_OF_Time Center) by normalizing an individual's measure to the mean and standard deviation of the comparison group (XX blank here). The directionality of scores was adjusted so that increased score values reflected increased anxiety-like emotionality. For example, decreased time in the open arms of the EPM was converted into positive deviation changes compared to group means, indicating increased anxiety-like emotionality. Second, each individual's *Z*-score was calculated within behavior tests (e.g., Z_EPM) by averaging the *Z*-scores of the behavior test measures (e.g., average of Z_EPM_Time Open and Z_EPM_%CrossesOpen). Third, an overall *Z*-score (anxiety-like emotionality) was obtained for each animal by averaging values across behavioral tests (e.g., average of Z_EPM and Z_OF). A graph showing effects of sex-related factors on anxiety-like emotionality *Z*-scores is included in Additional file 1: Figure S1. The overall Z-anxiety score for each animal could then be compared to gene expression using the Pearson correlation. The Benjamini-Hochberg method [49] was used with 5% false discovery rate; correlations were performed separately for GABA-related genes and serotonin/dopamine-related genes, and we controlled for the number of genes in each category (16 and 14, respectively).

### Heatmap visualization of gene expression results

We used the matrix2png online software to create expression heatmaps [51] for genes investigated and also for *Sst*, *Gad67*, and *Gad65* (reported in [13]). Expression for each main factor was expressed as the 'male’ phenotype divided by the female phenotype (i.e., sex chromosome complement, XY^-^/XX; gonadal sex, testes/ovaries; activational, testosterone/blank). If expression of a gene was higher in the male phenotype, the heatmap codes the color as red; if expression of a gene was higher in the female phenotype, the heatmap codes the color as blue.

### Gene network analysis

As was performed for heatmap visualization of gene expression results, we included expression values for *Sst*, *Gad67*, and *Gad65* (reported in [13]). The correlation strength was measured using the Pearson correlation coefficient raised by power 3 (i.e., |*r*|^3^) so as to enhance the signal from high correlation while penalizing those small correlations which could be introduced by random noise. Global graph properties are measured by density, clustering coefficient, and assortativity. *Density* measures how many edges are present in a network compared to the maximum possible number of edges:

$$\mathrm{Density}=\mathrm{mean}\left(k_{i}\right)/\left(n-1\right)$$

where *k*_*i*_ denotes the connectivity of node *i*.

*Global clustering coefficient* measures the extent to which nodes in a network tend to cluster together. When a node has *n* neighbors, there are at most *n*(*n - 1*) */ 2* edges that can exist between them (when every neighbor of the node is connected to every other neighbor). The local clustering coefficient of this node indicates the fraction of allowable edges that exist [52], and it is calculated as below in the context of the weighted network:

$${\mathrm{ClusterCoef}}_{i}\frac{\sum_{j\neq i}\sum_{k\neq i,j}w_{\mathrm{ij}}w_{\mathrm{jk}}w_{\mathrm{ki}}}{{\left(\sum_{j\neq i}w_{\mathrm{ij}}\right)}^{2}-\sum_{j\neq i}{\left(w_{\mathrm{ij}}\right)}^{2}}$$

*w*_*ij*_ represents the weight of edge between nodes *i* and *j* and the global clustering coefficient is calculated by averaging the local clustering coefficients for all nodes in the network.

*Assortativity* quantifies the likelihood that an edge will connect two nodes of similar degree, in other words, a preference for high-degree nodes to be connected to other high-degree nodes (here, the assortativity measure would be greater than 0). If a network has disassortativity (the assortativity measure is less than 0), high-degree nodes are more likely to be connected to low-degree nodes [53]. The assortativity value *r* for a network with *M* edges, connecting nodes of degrees *j* and *k*, is defined as

$$r=\frac{M^{-1}\sum_{i=1}^{m}j_{i}k_{i}-{\left[M^{-1}\sum_{i=1}^{m}\frac{1}{2}\left(j_{i}+k_{i}\right)\right]}^{2}}{M^{-1}{\sum_{i=1}^{m}\frac{1}{2}\left(j_{i}^{2}+k_{i}^{2}\right)-\left[M^{-1}\sum_{i=1}^{m}\frac{1}{2}\left(j_{i}+k_{i}\right)\right]}^{2}}$$

To compare global network properties between networks, the *p* value is calculated by randomly assigning mice to groups and determining how often the global graph properties between the two groups differ by chance; the permutation is repeated 1,000 times. These graph measures were calculated using igraph package from R CRAN. Paired networks for each main effect were visualized by the graphical software Cytoscape.

## Results

### Effects of sex-related factors on GABA-related genes

Refer to Table 1 for summary of statistical results and Additional file 2: Figure S2 for graphical representations of all GABA-related genes separated into all eight experimental groups. Note that we examined gene expression in all mice under chronic stress conditions.

**Table 1:** Statistical values associated with GABA-related gene expression in the frontal cortex of FCG mice

	**Gene**	**Sex chromosome**	**Gonadal sex**	**Activational**	**Significant interactions**
Dendritic-targeting GABA interneuron markers	*Vip*	*F* = 4.91; *df* = 1;	*F* = 0.32; *df* = 1;	*F* = 0.57; *df* = 1;	
		*p* < 0.03	*p* > 0.50	*p* > 0.45	
	*Calb1*	***F*** **= 6.66; *df*** **= 1;**	*F* = 0.02; *df* = 1;	***F*** **= 8.22; *df*** **= 1;**	Gonadal × Activational:
		***p*** **< 0.015**	*p* > 0.80	***p*** **= 0.005**	*F* = 9.09; *df* = 3; *p* < 0.005
	*Cst*	*F* = 1.51; *df* = 1;	*F* = 0.001; *df* = 1;	*F* = 3.26; *df* = 1;	Gonadal × Activational:
		*p* > 0.20	*p* > 0.95	*p* < 0.10	*F* = 8.625; *df* = 3; *p* < 0.005
	*Npy*	*F* = 1.11; *df* = 1;	*F* = 0.51; *df* = 1;	*F* = 1.93; *df* = 1;	
		*p* > 0.29	*p* > 0.45	*p* > 0.15	
Soma/interneuron-targeting GABA interneuron markers	*Cr*	*F* = 0.99; *df* = 1;	*F* = 0.26; *df* = 1;	*F* = 1.58; *df* = 1;	
		*p* > 0.32	*p* > 0.62	*p* > 0.20	
	*Cck*	*F* = 3.31; *df* = 1;	*F* = 0.53; *df* = 1;	*F* = 0.71; *df* = 1;	
		*p* < 0.08	*p* > 0.45	*p* > 0.40	
	*Pv*	*F* = 0.14; *df* = 1;	*F* = 0.01; *df* = 1;	*F* = 0.15; *df* = 1;	
		*p* > 0.70	*p* > 0.90	*p* > 0.65	
GABA-related genes	*Gat1*	***F*** **= 9.23; *df*** **= 1;**	*F* = 1.45; *df* = 1;	*F* = 2.33; *df* = 1	
		***p*** **< 0.005**	*p* > 0.20	*p* > 0.1	
	*Gabra1*	*F* = 0.13; *df* = 1;	*F* = 0.44; *df* = 1;	*F* = 0.56; *df* = 1;	
		*p* > 70	*p* > 0.50	*p* > 0.45	
	*Gabra2*	*F* = 0.38; *df* = 1;	*F* = 0.003; *df* = 1;	*F* = 0.64; *df* = 1;	
		*p* > 0.50	*p* > 0.95	*p* > 0.40	
	*Gabra5*	*F* = 0.02; *df* = 1;	*F* = 0.77; *df* = 1;	*F* = 3.47; *df* = 1;	
		*p* > 0.90	*p* > 0.35	*p* < 0.1	
BDNF-related genes	*Bdnf*	*F* = 0.40; *df* = 1;	*F* = 0.01; *df* = 1;	*F* = 1.72; *df* = 1;	Gonadal × Activational:
		*p* > 0.50	*p* > 0.90	*p* > 0.15	*F* = 4.84; *df* = 3; *p* < 0.04
	*Trkb*	***F*** **= 8.27; *df*** **= 1;**	*F* = 0.18; *df* = 1;	*F* = 1.62; *df* = 1;	
		***p*** **= 0.005**	*p* > 0.60	*p* > 0.20	

Numbers in bold indicate comparisons that were significant at 5% false discovery rate with correction for the 13 genes examined.

#### Markers of dendritic-targeting GABA interneurons

**Figure 1:**
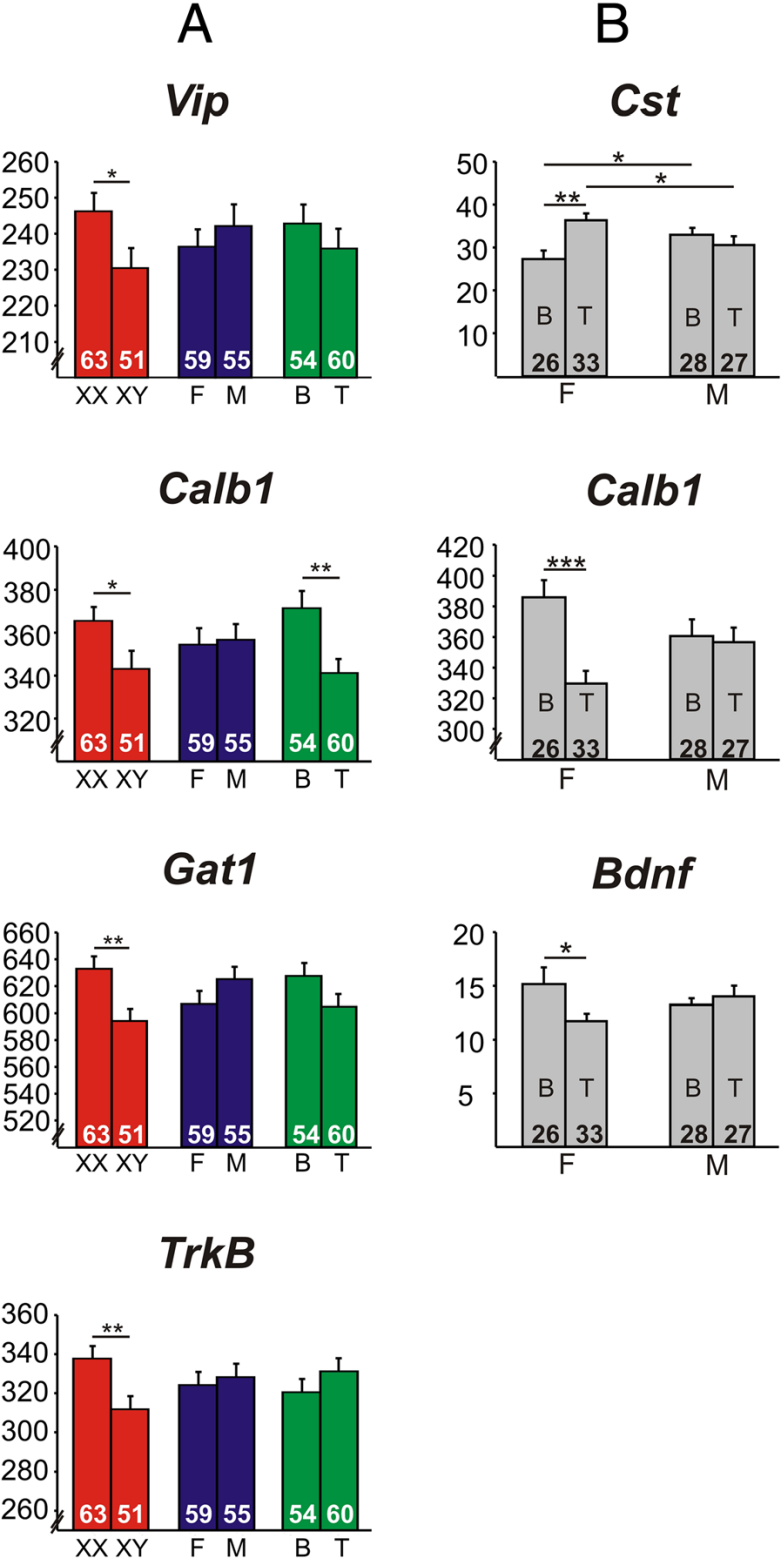
**Effects of sex-related factors on expression of GABA-related genes. (A)** GABA-related genes exhibiting significant main effects of sex chromosome complement (*Vip*, *Calb1*, *Gat1*, *Trkb*), gonadal sex (none), and/or circulating testosterone (*Calb1*). **(B)** GABA-related genes with significant interactions of sex-related factors. There was a significant interaction of gonadal sex and circulating testosterone on expression of *Cst*, *Calb1*, and *Bdnf. Numbers* at the base of *bars* indicate *N*. ***p < 0.001, **p < 0.01, *p < 0.05; *T* testosterone, *B* blank, *F* gonadal female, *M* gonadal male.

We observed an effect of sex chromosome complement on expression of *Vip* and *Calb1*, with lower expression of both genes in XY^-^ mice (Figure 1A). There was also a main effect of circulating testosterone on *Calb1* (Figure 1A) and an interaction of gonadal sex and circulating testosterone (Figure 1B); testosterone exposure decreased *Calb1* expression only in gonadal females. There was a significant interaction of gonadal sex and circulating testosterone on expression of *Cst* (Figure 1B). *Post hoc* analysis revealed an intricate picture of how organizational and activational effects of hormones affect *Cst* expression: (1) mice with ovaries during development and testosterone-treated in adulthood had higher *Cst* levels than blank-treated mice; (2) when gonadal males and females were treated with blank capsules after gonadectomy, gonadal males had higher *Cst* expression; and conversely, (3) when gonadal males and females were testosterone-treated, gonadal females had higher *Cst* expression. There were no effects of sex-related factors on *Npy* gene expression.

#### Markers of soma/interneuron-targeting GABA interneurons

We found that sex-related factors had no effect on expression of *Cr*, *Cck*, or *Pv*.

#### Other GABA-related genes

We found a significant effect of sex chromosome complement on expression of *Gat1* (Figure 1A), with lower expression in XY^-^ mice. There were no significant effects of sex-related factors on expression of the GABA A subunits, *Gabra1*, *Gabra2*, or *Gabra5*.

#### BDNF-related genes

There was a main effect of sex chromosome complement on expression of the BDNF receptor *Trkb* (Figure 1A), with lower expression in XY^-^ mice. We observed no main effects on *Bdnf* expression, but there was a significant interaction of gonadal sex and circulating testosterone (Figure 1B); gonadal females treated in adulthood with blank had higher *Bdnf* levels compared to those treated with testosterone.

We controlled the false positive rate using the Benjamini-Hochberg method [49] and corrected for the number of GABA-related genes examined. At 5% false discovery rate, there was a significant effect of genetic sex on *Calb1*, *Gat1*, and *Trkb* expression, an effect of circulating testosterone on *Calb1* expression, and interactions between gonadal sex and circulating testosterone on *Calb1* and *Cst* expression. These findings suggest that sex chromosome complement is the main factor influencing expression of the GABA-related genes examined in the frontal cortex under conditions of chronic stress exposure. Based on findings in humans indicating reduced expression of GABA-related genes in MDD [8,10-12] and on genetic manipulations in mice [54], results were in a direction suggesting that XY^-^ mice would be more vulnerable to elevated anxiety/depressive-like behavior.

### Effects of sex-related factors on serotonin- and dopamine-related genes

Refer to Table 2 for summary of statistical results and Additional file 3: Figure S3 for graphical representations of all serotonin- and dopamine-related genes separated into all eight experimental groups. Note that we examined gene expression in all mice under chronic stress conditions.

**Table 2:** Statistical values associated with serotonin- and dopamine-related gene expression in the frontal cortex

	**Gene**	**Sex chromosome**	**Gonadal sex**	**Activational**	**Significant interactions**
Serotonin and dopamine receptors	*Htr1a*	***F*** **= 4.77; *df*** **= 1;**	***F*** **= 3.65; *df*** **= 1;**	***F*** **= 20.58; *df*** **= 1;**	Gonadal × Activational:
		***p*** **< 0.04**	***p*** **< 0.10**	***p*** **< 0.001**	*F* = 11.92; *df* = 3; *p* < 0.001
	*Htr2a*	*F* = 0.22; *df* = 1;	***F*** **= 4.32; *df*** **= 1;**	*F* = 0.11; *df* = 1;	
		*p* > 0.60	***p*** **= 0.040**	*p* > 0.70	
	*Htr2c*	***F*** **= 6.29; *df*** **= 1;**	*F* = 2.16; *df* = 1;	*F* = 1.88; *df* = 1;	
		***p*** **< 0.02**	*p* > 0.10	*p* > 0.15	
	*Drd1a*	***F*** **= 4.01; *df*** **= 1;**	*F* = 0.049; *df* = 1;	*F* = 0.13; *df* = 1;	Gonadal × Activational:
		***p*** **< 0.05**	*p* > 0.80	*p* > 0.70	*F* = 2.86; *df* = 3; *p* < 0.045
cAMP/PKA pathway	*Adcy1*	*F* = 1.28; *df* = 1;	***F*** **= 4.59; *df*** **= 1;**	*F* = 0.55; *df* = 1;	Sex chromosome × Activational:
		*p* > 0.25	***p*** **< 0.04**	*p* > 0.45	*F* = 2.70; *df* = 3; *p* < 0.05
					Sex chromosome × Gonadal × Activational: *F* = 2.94; *df* = 7; *p* < 0.01
	*Adcy2*	***F*** **= 3.32; *df*** **= 1;**	*F* = 2.35; *df* = 1;	***F*** **= 8.83; *df*** **= 1;**	
		***p*** **= 0.071**	*p* > 0.10	***p*** **< 0.005**	
	*Adcy5*	***F*** **= 5.73; *df*** **= 1;**	*F* = 2.94; *df* = 1;	*F* = 0.062; *df* = 1;	Genetic × Activational:
		***p*** **< 0.02**	*p* = 0.089	*p* > 0.75	*F* = 2.68; *df* = 3; *p* = 0.11
					Gonadal × Activational:
					*F* = 3.63; *df* = 3; *p* < 0.02
	*Adcy7*	***F*** **= 5.37; *df*** **= 1;**	*F* = 0.94; *df* = 1;	*F* = 0.08; *df* = 1;	
		***p*** **< 0.025**	*p* > 0.30	*p* > 0.75	
	*Cdk5*	***F*** **= 2.93; *df*** **= 1;**	*F* = 1.01; *df* = 1;	***F*** **= 2.90; *df*** **= 1;**	Gonadal × Activational:
		***p*** **= 0.09**	*p* > 0.30	***p*** **= 0.091**	*F* = 2.67; *df* = 3; *p* = 0.051
AKT pathway	*Akt1*	***F*** **= 3.75; *df*** **= 1;**	*F* = 0.61; *df* = 1;	***F*** **= 5.99; *df*** **= 1;**	Sex chromosome × Gonadal:
		***p*** **= 0.056**	*p* > 0.40	***p*** **< 0.02**	*F* =2.53; *df* = 3; *p* = 0.11
					Sex chromosome × Activational:
					*F* = 4.82; *df* = 3; *p* < 0.005
					Gonadal × Activational:
					*F* = 4.16; *df* = 7; *p* < 0.01
	*Akt2*	*F* = 0.89; *df* = 1;	***F*** **= 4.64; *df*** **= 1;**	*F* = 2.10; *df* = 1;	
		*p* > 0.30	***p*** **< 0.035**	*p* > 0.10	
	*Akt3*	***F*** **= 3.57; *df*** **= 1;**	***F*** **= 7.16; *df*** **= 1;**	***F*** **= 4.72; *df*** **= 1;**	Sex chromosome × Activational:
		***p*** **= 0.062**	***p*** **< 0.01**	***p*** **< 0.035**	*F* = 2.64; *df* = 3; *p* = 0.11
					Gonadal × Activational:
					*F* = 6.53; *df* = 3; *p* < 0.001
Other serotonin- and dopamine-related genes	*App*	***F*** **= 6.56; *df*** **= 1;**	*F* = 1.43; *df* = 1;	*F* = 0.22; *df* = 1;	Gonadal × Activational:
		***p*** **< 0.015**	*p* > 0.20	*p* > 0.60	*F* = 2.17; *df* = 3; *p* = 0.096
					Sex chromosome × Gonadal ×
					Activational: *F* = 2.62; *df* = 7; *p* = 0.11
	*Pdyn*	*F* = 0.29; *df* = 1;	*F* = 0.38; *df* = 1;	*F* = 2.09; *df* = 1;	
			*p* > 0.55	*p* > 0.50	*p* > 0.15

Numbers in bold indicate comparisons that were significant at 5% false discovery rate with correction for the 14 genes examined. *cAMP* cyclic adenosine monophosphate, *PKA* protein kinase A.

#### Serotonin and dopamine receptors

**Figure 2:**
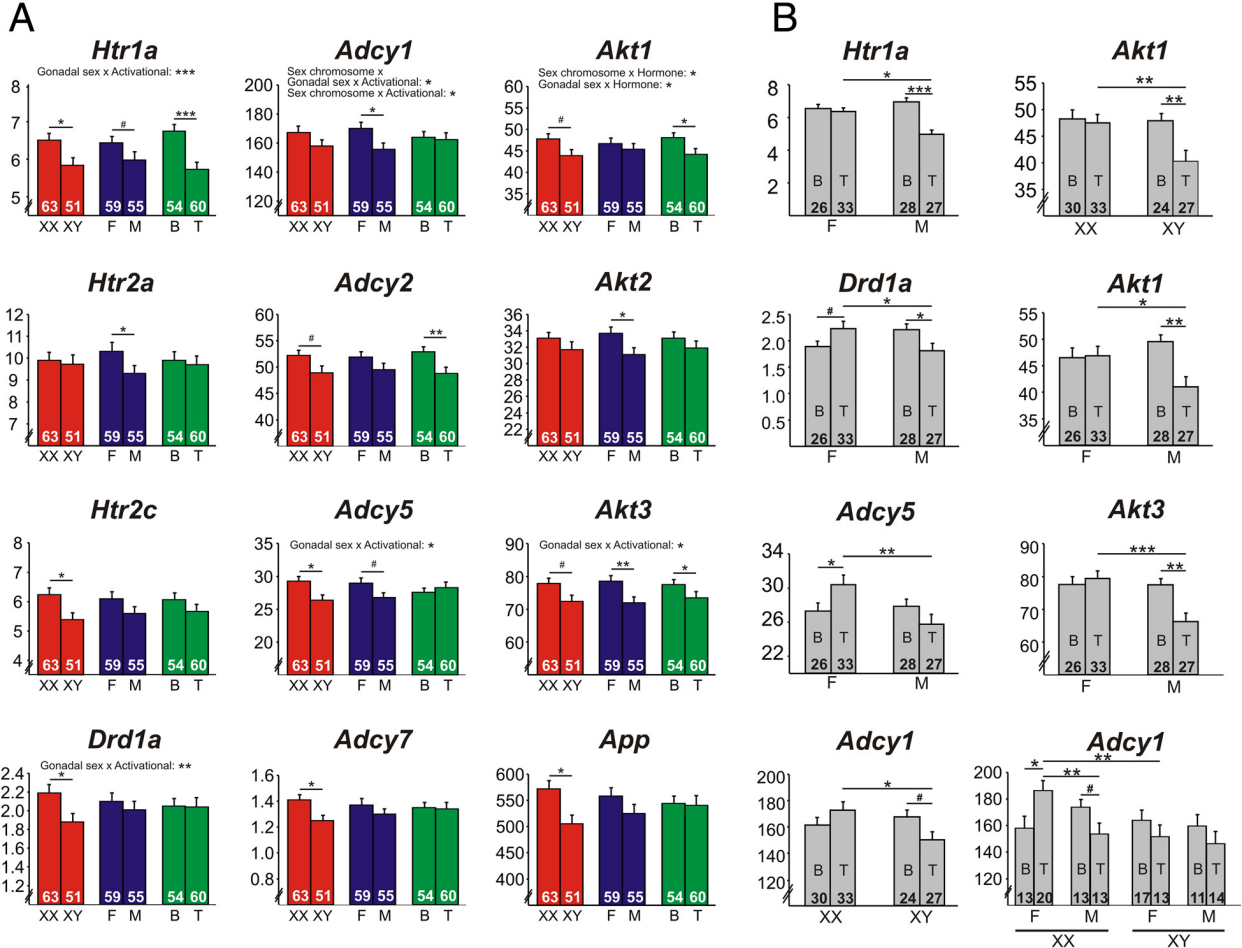
**Effects of sex-related factors on expression of serotonin (5HT)- and dopamine (DA)-related genes. (A)** 5HT- and DA-related genes exhibiting significant main effects of sex chromosome complement (*Htr1a*, *Htr2c*, *Drd1a*, *Adcy1*, *Adcy2*, *Adcy5*, *Adcy7*, *Akt1*, *Akt3*, *App*), gonadal sex (*Htr1a*, *Htr2a*, *Adcy1*, *Adcy5*, *Akt2*, *Akt3*), and/or circulating testosterone (*Htr1a*, *Adcy2*, *Akt1*, *Akt3*). **(B)** 5HT- and DA-related genes with significant interactions of gonadal sex and circulating testosterone (*Htr1a*, *Drd1a*, *Adcy5*, *Akt1*, *Akt3*), sex chromosome complement and circulating testosterone (*Adcy1*, *Akt1*), or interaction of all three sex-related factors (*Adcy1*). *Numbers* at the base of *bars* indicate *N*. ***p < 0.001, **p < 0.01, *p < 0.05, #*p* < 0.1; *T* testosterone, *B* blank, *F* gonadal female, *M* gonadal male.

There was a main effect of sex chromosome complement on *Htr2c*, with lower expression in XY^-^ compared to XX mice (Figure 2A). There was a main effect of gonadal sex on expression of *Htr2a*, with higher expression in gonadal females compared to gonadal males (Figure 2A). There were main effects of sex chromosome complement (XY^-^ < XX) and circulating testosterone (testosterone > blank) on *Htr1a* expression (Figure 2A) and a significant interaction of circulating testosterone and gonadal sex. Circulating testosterone caused a decrease in *Htr1a* expression in gonadal males, but not gonadal females, and when both gonadal males and females were treated with testosterone, *Htr1a* expression was lower in the gonadal males (Figure 2B). For *Drd1a*, there was a main effect of sex chromosome complement (XY^-^ < XX; Figure 2A) and a significant interaction of circulating testosterone and gonadal sex. Circulating testosterone increased *Drd1a* expression in gonadal females, but decreased expression in gonadal males; additionally, when both gonadal males and females were treated with testosterone, *Drd1a* expression was lower in gonadal males (Figure 2B).

#### Signal transduction-related genes

##### cAMP/PKA pathway

For *Adcy1*, there was a main effect of gonadal sex (ovaries > testes; Figure 2A), a significant interaction of sex chromosome complement and circulating testosterone (circulating testosterone lowered *Adcy1* expression in XY^-^, but not XX mice; Figure 2B), and a significant interaction of all three sex-related factors. Circulating testosterone increased *Adcy1* expression in XX gonadal females and decreased *Adcy1* expression in XX gonadal males; there was no effect of testosterone in XY^-^ gonadal females or males (Figure 2B). There was a main effect of circulating testosterone on *Adcy2* expression, with circulating testosterone causing a decrease in *Adcy2* expression (Figure 2A). For *Adcy5*, there was a main effect of sex chromosome complement (XY^-^ < XX; Figure 2A) and a significant interaction of circulating testosterone and gonadal sex. Circulating testosterone increased *Adcy5* expression in gonadal females, but not gonadal males, and when both gonadal males and females were treated with testosterone, *Adcy5* was lower in gonadal males (Figure 2B). There was a main effect of sex chromosome complement on *Adcy7* expression, with lower expression in XY^-^ mice (Figure 2A). There were no main effects on *Cdk5* expression; however, there was a trend for an interaction between circulating testosterone and gonadal sex. Testosterone decreased *Cdk5* expression in gonadal males only, and when gonadal males and females were treated with testosterone, *Cdk5* expression was lower in gonadal males (Figure 2B).

##### AKT pathway-related genes

For *Akt1*, there was a main effect of circulating testosterone on expression (Figure 2A) and interactions between sex chromosome complement and circulating testosterone as well as between gonadal sex and circulating testosterone. Testosterone decreased *Akt1* expression in XY^-^, but not XX mice; when XX and XY^-^ mice were both treated with testosterone, *Akt1* expression was lower in XY^-^ mice (Figure 2B). Additionally, testosterone decreased *Akt1* expression in gonadal males, but not gonadal females; when gonadal males and females were treated with testosterone, *Akt1* expression was lower in gonadal males (Figure 2B). There was a main effect of gonadal sex on expression of *Akt2*, with lower expression in gonadal males (Figure 2A). For *Akt3*, there were main effects of circulating testosterone and gonadal sex (Figure 2A) and a significant interaction of circulating testosterone and gonadal sex. Circulating testosterone decreased *Akt3* expression only in gonadal males; when both gonadal males and females were treated with testosterone, *Akt3* expression was lower in gonadal males (Figure 2B).

#### Other serotonin- and dopamine-related genes

There was a main effect of sex chromosome complement on *App* expression, with lower expression in XY^-^ mice (Figure 2A). We found no effects of sex-related factors on *Pdny* expression.

All significant results found using uncorrected *p* values remained significant after performing a Benjamini-Hochberg correction [49] at 5% false discovery rate for the number of serotonin/dopamine-related genes examined. Similar to our findings for GABA-related genes, sex chromosome complement is the main factor influencing expression of the serotonin/dopamine-related genes examined in the frontal cortex under conditions of chronic stress exposure. Here, however, several genes were influenced by the interaction of gonadal sex and adult circulating testosterone, with testosterone treatment causing a decrease in gene expression only in gonadal males.

### Correlation between gene expression and anxiety-like behavior

Refer to Table 3 for summary of genes positively or negatively correlated with anxiety-like behavior (after Benjamin-Hochberg correction and 5% false discovery rate) [49] and Additional file 4: Table S1 and Additional file 5: Table S2 for statistical values associated with each correlation test. We first searched for significant correlations between anxiety-like behavior and gene expression in all mice combined and found that *Trkb*, *Htr2c*, *Adcy5*, and *App* were negatively correlated with anxiety-like behavior. We next examined potential correlations between gene expression and anxiety-like behavior after splitting mice by each main sex-related factor (XX, XY, gonadal males, gonadal females, blank-treated, testosterone-treated) to search for differential correlation patterns. Interestingly, patterns of correlation between gene expression and anxiety-like behavior seem to vary by sex-related factors (i.e., XX versus XY^-^, gonadal males versus gonadal females). For instance, while *Pdyn* is the only gene positively correlated with anxiety-like behavior in XX mice, *Htr1a*, *Cdk5*, *Adcy2*, *Akt1*, *Akt2*, and *Akt3* positively correlate with anxiety-like behavior in XY^-^ mice. Another interesting observation based on this correlation analysis is that gonadal males and gonadal females exhibit opposite patterns of correlation for many genes; while *Adcy1*, *Adcy2*, *Akt1*, and *Akt3* are negatively correlated with anxiety-like behavior in gonadal females, these same genes are positively correlated with anxiety-like behavior in gonadal males. The correlation patterns based on hormone treatment were fairly week, with only *Htr2c* and *App* being negatively correlated with anxiety-like behavior in testosterone-treated mice.

**Table 3:** Genes correlated with anxiety-like behavior

**Correlation**	**Genes**
(+) correlation with anxiety-like behavior overall	None
(-) correlation with anxiety-like behavior overall	*Trkb*, *Htr2c*, *Adcy5*, *App*
(+) correlation with anxiety-like behavior in XX	*Pdyn*
(-) correlation with anxiety-like behavior in XX	*Htr2c*, *Adcy5*, *App*,
(+) correlation with anxiety-like behavior in XY	*Htr1a*, *Cdk5*, *Adcy2*, *Akt1*, *Akt2*, *Akt3*
(-) correlation with anxiety-like behavior in XY	None
(+) correlation with anxiety-like behavior in gonadal females	None
(-) correlation with anxiety-like behavior in gonadal females	*Cst*, *Drd1a*, *Htr2c*, ***Adcy1***, ***Adcy2***, *Adcy5*, *Adcy7*, ***Akt1***, ***Akt3***, *App*
(+) correlation with anxiety-like behavior in gonadal males	*Htr1a*, ***Adcy1***, ***Adcy2***, *Cdk5*, ***Akt1***, ***Akt3***
(-) correlation with anxiety-like behavior in gonadal males	None
(+) correlation with anxiety-like behavior in blank-treated	None
(-) correlation with anxiety-like behavior in blank-treated	None
(+) correlation with anxiety-like behavior in T-treated	None
(-) correlation with anxiety-like behavior in T-treated	*Htr2c*, *App*

Genes with a negative correlation with anxiety-like behavior in gonadal females and positive correlation with anxiety-like behavior in gonadal males are indicated in bold.

### Heatmap representation of gene expression by main sex-related factor in FCG mice

**Figure 3:**
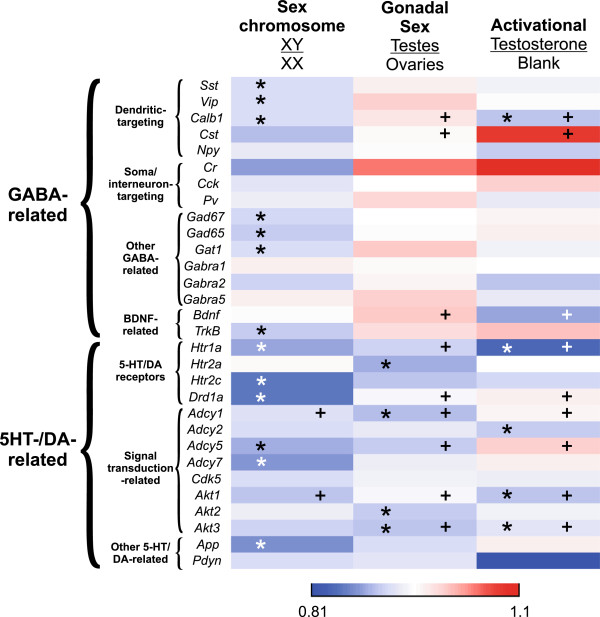
**Heatmap representation of gene expression results.** Expression for each main factor is expressed as the male phenotype divided by the female phenotype (i.e., sex chromosome, XY/XX; gonadal sex, testes/ovaries; activational, testosterone/blank). *Red* indicates that the gene was expressed at higher levels in the male phenotype; *blue* indicates that the gene was expressed at higher levels in the female phenotype. The *asterisk* indicates significant main effect of that factor; the *plus sign* indicates significant interaction involving that factor.

To summarize the qPCR data and extract patterns of influence for sex-related factors on gene expression, we visualized results for all genes using a heatmap. We included results for three GABA-related genes (*Sst*, *Gad65*, and *Gad67*) from a previous report using the same mice [13]. Not surprisingly, based on the pattern of results for GABA-related, serotonin/dopamine-related, and signal transduction pathway-related genes, the pattern of reduced expression in XY^-^ mice compared to XX was the most striking feature of the graph (indicated by the blue color of most genes for the sex chromosome comparison) (Figure 3). Interestingly, the summarized results for gonadal sex suggested an overall pattern of higher expression of GABA-related genes (shown in red) and lower expression of serotonin/dopamine-related genes (shown in blue) in gonadal males (Figure 3). Note that most of these gonadal sex comparisons and those for activational effects did not yield significant results in the ANOVA analysis, but the pattern of results is still apparent in the heatmap representation. The results for activational effects of testosterone were mixed; expression of some genes was higher in testosterone-treated mice (shown in red), while other genes had higher expression in blank-treated mice (shown in blue) (Figure 3). Thus, it appears as though the general effect of male sex chromosome complement to decrease gene expression is only partially reversed by the effects of male gonadal sex and circulating testosterone. Together, these results indicate balancing roles between sex-related factors, with a potential dominant effect of male sex chromosome complement against male gonadal sex and circulating testosterone in controlling GABA/serotonin/dopamine-related gene expression.

### Gene network-based analysis

**Figure 4:**
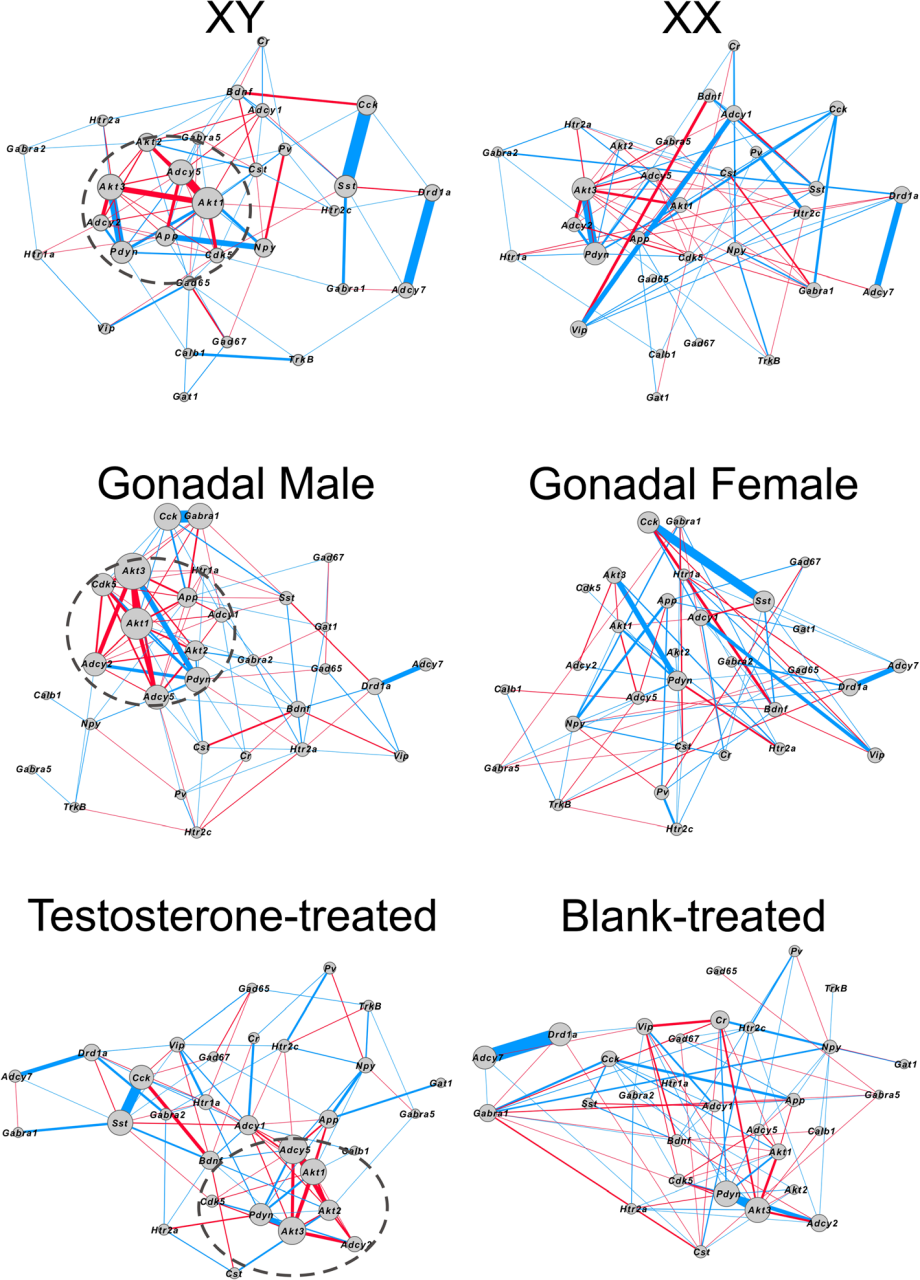
**Weighted gene co-expression networks of all GABA-, serotonin-, and dopamine-related genes.** Networks are split based on sex chromosome complement, gonadal sex, and hormone treatment. *Lines* connecting genes indicate co-expression of those two genes, with the weight of the line proportional to the level of co-expression. *Red lines* indicate positive correlation, while *blue lines* indicate negative correlation between genes. The size of each node indicates the degree (sum of the edge weights of all connections) for that gene. The male phenotypes (XY^-^, gonadal male, testosterone-treated) were used as reference networks due to their robust effect on expression, meaning that the female data (XX, gonadal female, blank-treated) was plotted on the network structure established by the respective male phenotype.

We next applied co-expression network analyses as an alternative approach to investigate the effects of sex-related factors on expression patterns. Two genes or transcripts are considered co-expressed if their patterns of expression are correlated across samples; this link has been shown to reflect shared function, through multiple potential biological pathways, including common regulatory pathways (e.g., hormone signaling, transcription factors) [55,56]. Weighted gene co-expression networks were constructed separately for each of the three main effects (XX versus XY^-^, gonadal female versus gonadal male, blank-treated versus testosterone-treated); nodes represent the genes investigated and edges represent the correlation magnitude of gene co-expression. The male phenotype (XY^-^, gonadal male, testosterone-treated) was used as the reference network due to its robust effect on differential expression (Figure 4), and this structure was applied to the data for each female phenotype (XY^-^, gonadal female, blank-treated). The male network for chromosomal sex and gonadal sex exhibited increased assortativity compared to the associated female networks (*p* < 0.03 for both comparisons; Table 4). Notably, the XX and gonadal female networks exhibit disassortativity (i.e., assortativity measures less than 0), indicating that in these networks, nodes with high degree are likely to be connected to nodes with low degree; the XY and gonadal male networks have assortativity measures greater than 0, indicating that high-degree nodes are likely to be connected to other high-degree nodes. Additionally, there was a trend for the male activational network (i.e., testosterone-treated) to exhibit increased density compared to the female activational network (*p* < 0.09; Table 4), indicating that the male activational network has more overall connections between nodes. It is important to note that the significant differences we see between networks are not driven by effects of other sex-related factors. For instance, the XX and XY^-^ networks both contain gonadal males, gonadal females, mice treated with blank, and mice treated with testosterone. Therefore, the only difference between the XX and XY^-^ networks is the sex chromosomal difference, and we can conclude that any significant differences we see between networks are driven by the sex chromosome difference. These statistical differences between networks were visually reflected in the male networks having more strong connections between genes (i.e., high correlation; indicated by thicker lines connecting genes) compared to the matched female networks (Figure 4). Notably, all three male networks (XY^-^ mice, gonadal males, and testosterone-treated mice) displayed a similar tightly linked co-expression module of signal transduction-related genes with high degree (i.e., hubness; indicated by the size of the node) that was not present in XX mice, gonadal females, or blank-treated mice (Figure 4). This core module includes *Akt1*, *Akt2*, *Akt3*, *Adcy2*, *Adcy5*, and *Pdyn* in XY^-^ mice, gonadal males, and testosterone-treated mice; the core module in XY^-^ mice and gonadal males also includes *Cdk5* and *App*.

**Table 4:** Statistical values associated with global network measures

**Global network property**	**XX**	**XY**	**Sex chromosome *p *** **value**	**Gonadal female**	**Gonadal male**	**Gonadal sex *p *** **value**	**Blank-treated**	**T-treated**	**Activational *p *** **value**
Density	0.016	0.022	0.213	0.016	0.022	0.142	0.017	0.022	0.081
Global clustering coefficient	0.037	0.050	0.334	0.044	0.056	0.297	0.035	0.049	0.213
Assortativity	-0.089	0.120	0.025	-0.084	0.124	0.027	-0.062	0.010	0.274

## Discussion

**Figure 5:**
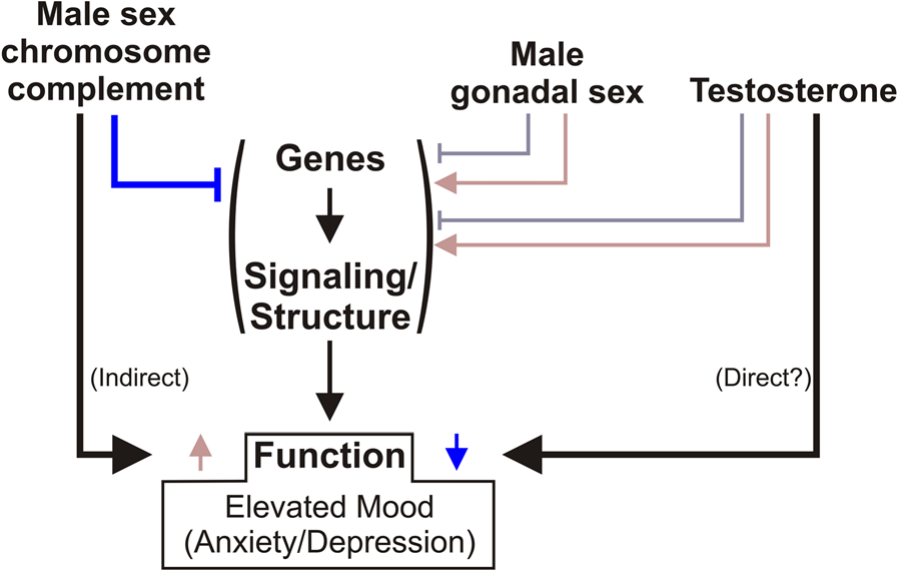
**Model representing the dynamic equilibrium controlling gene expression (i.e., structure) and associated anxiety-like behaviors (i.e., function).***Blue lines* indicate decreased gene expression or anxiety-like behavior and *red arrows* indicate increased gene expression or anxiety-like behavior; the *brightness of the color* indicates intensity of the effect, with *darker shades* indicating increased robustness of the effect.

Using the FCG mice, we examined the effect of sex chromosome complement, developmental gonadal sex, and circulating testosterone on frontal cortex expression of selected GABA-, serotonin-, and dopamine-related genes, after exposure to unpredictable chronic mild stress, as a validated translational model for depressive-like pathology. Overall, the most robust main effect was that of sex chromosome complement, which consistently caused lower gene expression in XY^-^ compared to XX mice (Figures 1 and 2). While we did see main effects of gonadal sex and circulating testosterone on gene expression, they were mostly in the context of interactions between these two main factors (Figures 1 and 2). Pearson correlation analysis between gene expression and anxiety-like behavior measures from the same mice revealed that gene-behavior correlation patterns vary by sex-related factors (XX versus XY^-^ and gonadal female versus gonadal male). Heatmap representation of the data confirmed the general pattern of sex chromosome complement-mediated decreased expression in XY^-^ mice (indicated by blue color in Figure 3) that was partially reversed by effects of male gonadal sex and circulating testosterone (indicated by red color in Figure 3). Finally, a gene co-expression network analysis indicated overall increased expression connectedness in male networks and suggested the presence of a core module of signal transduction-related genes in all three networks corresponding to the same male phenotype for each main factor (Figure 4). These results suggest that the effect of male sex chromosome complement on lowering expression of genes related to mood in humans serves to set up a structure (i.e., gene expression, signaling) that differs by sex chromosome complement and that is supportive of a 'disease state,’ as gene changes were often in pro-disease direction (i.e., 'indirect’ effect; Figure 5). This structure is then acted upon by male gonadal sex and circulating testosterone, which partially opposes the effects of male sex chromosome complement on gene expression (i.e., 'direct’ effect; Figure 5), together creating a dynamic biological equilibrium regulating gene expression (i.e., the structure) and adult anxiety-like behaviors (i.e., the function) (Figure 5). Notably, although circulating testosterone only partially reverses the effect of male sex chromosome complement on expression of genes related to mood regulation in humans, we previously reported that at the behavioral level, circulating testosterone more than compensated for the increased anxiety-like behavior caused by male sex chromosome complement [13] (Figure 5), consistent with normal males having lower anxiety-like behaviors than females. The surprising effect of sex chromosome complement on expression of GABA-, serotonin-, and dopamine-related genes establishes this as a critical and significant contributor to expression of mouse genes related to mood regulation in humans and associated sexual dimorphism.

### Sex chromosome complement as a contributing factor

As apparent in Figure 3, sex chromosome complement was the factor that exhibited the highest proportion of main effects on GABA-, serotonin-, and dopamine-related gene expression. Notably, all significant effects of sex chromosome complement were in the same direction, with XY^-^ mice having lower gene expression compared to XX mice. Our current findings are consistent with a proposed hypothesis regarding sex chromosome complement and gene expression [57]. It has also been suggested that X inactivation may result in a large heterochromatic sink in XX cells that could influence epigenetic processes on all chromosomes [58]. If the cell has a limited supply of epigenetic machinery, and much of this machinery is being used to inactivating an X, these XX cells would have a lower supply available for autosomal gene regulation, the end result being XX cells having globally higher expression of genes sensitive to heterochromatizing factors (for example, genes near heterochromatic regions or under epigenetic regulation). There are several additional mechanisms that could underlie effects of sex chromosome complement on gene expression. Structurally, sex chromosomes differ between males and females in the presence of a Y chromosome in males, dosage of X chromosomes, and presence of a paternal X imprint in females only. Additional sex chromosome complement differences arise during development; for instance, X inactivation occurs only in females, and due to random X inactivation, female tissues are mosaics, with about half of cells expressing an active maternal X and the other half expressing an active paternal X. It has also been reported that some X chromosome genes in females escape X-inactivation; however, the number of X-escapees is low in mice (approximately 3%) [59,60].

### Interaction of gonadal sex and circulating testosterone

Here, we found that expression of a number of genes varied based on gonadal sex by circulating testosterone interactions. Although not true for all significant interactions, most followed a similar pattern, with testosterone treatment causing a decrease in gene expression only in gonadal males (Figures 1B and 2B). These results support the well-established concept that the organizational effects of gonadal hormones during development create differences in how the adult brain responds to testosterone (e.g., [30]; reviewed in [34]). These results also strengthen the important fact that gonadal males and females respond differently to adult hormone levels, indicating that hormone treatments in humans may yield different results in men and women.

### How can these results inform risk for psychopathology in humans?

#### GABA-related genes

XY^-^ mice, regardless of gonadal sex or circulating hormones, exhibited reduced expression of genes coding for markers of dendritic-targeting GABA interneurons (*Sst*, *Vip*, *Calb1*) and for GABA-synthesizing enzymes (*Gad67/65*) (Figure 1A; [13]). Interestingly, XY^-^ mice also displayed elevated anxiety-like behaviors compared to XX animals [13]. These molecular findings partly mimic changes observed in human subjects affected with MDD (see the 'Background’ section and [8,10-12]), supporting the interpretation of a causal/contributing role of reduced dendritic-targeting interneuron-mediated inhibition to the respective behavioral phenotypes. Notably, the results that we report here for *Calb1* (XY^-^ < XX, testosterone-treated < blank-treated) are consistent with previous reports for *Calb1* gene expression in the frontal cortex of FCG mice (XY^-^ < XX, effect of estrogen receptor alpha signaling [61,62]). A previous study from our group found reduced *TRKB* expression in the sgACC of *postmortem* brains of patients with MDD [11], which is consistent with our current finding of reduced *Trkb* (Figure 1A) and increased anxiety-like behavior in XY^-^ mice and negative correlation of *Trkb* with anxiety-like behavior.

#### Serotonin/dopamine-related genes

Htr1a controls serotonin presynaptic release and contributes to postsynaptic signaling, and reduced or lack of expression is associated with increased anxiety-like behaviors [63,64]. Htr2a/2c have been consistently associated with mood-related phenotypes and are targeted for the development of therapeutic compounds [65]. Accordingly, reduced *Htra1* and *Htr2c* expressions in XY mice are consistent with the increased depressive-like behaviors observed in that group [13] and together suggests a sexual dimorphism aspect to the low serotonin hypothesis of depression [1]. Not consistent with these previous findings [63,64], we found that *Htr1a* was positively correlated with anxiety-like behavior in XY mice and gonadal males. However, our finding that *Htr2c* expression was negatively correlated with anxiety-like behavior overall and in gonadal females is consistent with these previous reports (Table 3). A previous study reported that *Adcy5* knockout mice have poor stress coping responses [66], a finding that is consistent with our current finding that chronically stressed XY^-^ mice have lower *Adcy5* gene expression (Figure 2A) and increased anxiety-like behavior compared to XX mice [13]. Interestingly, we also found a negative correlation of *Adcy5* with anxiety-like behavior (Table 3). Here, we also report that XY^-^ mice have lower expression of *Adcy7* compared to XX mice (Figure 2A) and a negative correlation between *Adcy7* expression and anxiety-like behavior in gonadal females. This result is not consistent with reports that decreased *Adcy7* results in decreased depressive-like behavior [67] and with previous finding of elevated *ADCY7* in the brains of MDD subjects [68], suggesting that the decreased *Adcy7* in our XY^-^ mice does not underlie their elevated anxiety-like behavior*.* We found that *Akt1* was reduced in XY^-^ mice but only when treated in adulthood with testosterone (Figure 2B). Interestingly, one report found reduced *AKT1* activity in the brains of depressed suicide victims [69]. In our correlational analysis, there were a number of genes that were negatively correlated in gonadal females, but positively correlated in gonadal males; this suggests that gonadal males and females may respond differently to differences in expression of these genes or that additional factors moderate these behaviors (e.g., protein expression or independent factors).

Together, these findings of reduced GABA-, serotonin-, and dopamine-related gene expression in XY^-^ mice show that sex chromosome complement plays a role in programming expression of these systems to be lower in individuals with male sex chromosome complement (often in pro-disease direction). This result is surprising, as women are more vulnerable to mood disorders, and suggests that other factors (gonadal sex or circulating testosterone) in normal males may compensate for the sex chromosome complement effect on gene expression. Even though our results did not clearly identify this factor, the heatmap results show partial, non-significant compensation by a number of genes due to male gonadal sex and testosterone (Figure 3). Of note, our previous study in these same FCG mice found that circulating testosterone potently decreased anxiety-like behaviors [13]; thus, future studies will aim to identify genes and proteins whose expression is altered in anti-disease directions by circulating testosterone treatment.

### Limitations

Although the FCG mice provide a valuable tool for parsing the relative contributions of sex chromosome complement, gonadal sex, and circulating hormone to sexual dimorphism, there are limitations to this model that must be considered. First, XY^-^ mice are not identical to wild-type XY mice as the Y chromosome of XY^-^ mice lacks the *Sry* gene, but note that when *Sry* is added back to XY^-^ mice on an autosome, these XY^-^*Sry* male mice are fertile and display no differences from wild-type testosterone levels [36,37,70]. Second, normal gene expression could be disrupted after autosomal *Sry* transgene insertion. Third, it has been suggested that XY^-^ females stop cycling early. Our design precludes this possibility from having an impact, however, since all mice were gonadectomized 10 weeks prior to sacrifice and gene expression analysis. Fourth, in addition to its developmental role in gonad differentiation, *Sry* is also expressed in the adult mouse brain [71]; thus, it is not possible to distinguish indirect effects of *Sry* in gonad differentiation (i.e., organizational) from direct effect based on adult *Sry* gene expression in FCG mice. Other potential limitations to the design of this study are that we do not have a group of mice that was gonadectomized with estradiol and progesterone replacement and we do not examine gene expression under baseline (i.e., non-stress) conditions. Evidence suggests that testosterone reduces anxiety-like behaviors [72-75]; on the other hand, estradiol exposure has been reported to both increase and decrease anxiety-like behavior [76-78]. We chose to perform testosterone rather than estradiol replacement based on the more consistent reported results for testosterone on anxiety-like behavior [72-75]. The study design employed here (baseline behavior testing, followed by 8 weeks of chronic stress and stress-induced behavior testing, and sacrifice) was employed due to our interest in identifying genes that may vary by sex in the context of chronic stress exposure. Based on this design, however, we are unable to discern whether effects that we see are *due* to chronic stress since we did not examine gene expression under non-stressed conditions. This design was partially dictated by experimental constraints on the number of mice that can reasonably be used in a behavior/chronic stress study. Future studies will examine gene expression in FCG mice under non-stress conditions and will compare mice gonadectomized and implanted with blank or estradiol-filled capsules. Finally, we investigated only selected genes within the respective pathways. Large-scale expression analyses are needed to assess the full extent of the impact of the respective factors.

## Conclusions

These studies provide evidence for a robust effect of XY sex chromosome complement to decrease expression of genes related to fast inhibitory GABA signaling and neuromodulatory serotonin and dopamine signaling. Combined with (1) our previous results indicating elevated anxiety-like behaviors in XY mice, (2) evidence for involvement of these systems in the pathology of mood disorders, and (3) concordant directions of gene changes, these results provide molecular leads to investigate the biological bases of the sexual dimorphism observed in mood disorders and specifically identify male XY sex chromosome complement as a pro-disease factor.

## Abbreviations

Adcy: Adenylate cyclase; App: Amyloid precursor protein; BDNF: Brain-derived neurotrophic factor; cAMP: Cyclic adenosine monophosphate; Cck: Cholecystokinin; Cdk5: Cyclin-dependent kinase 5; Cr: Calretinin; Cst: Cortistatin; Drd1a: Dopamine receptor D1a; FCG: Four Core Genotypes; GABA: Gamma-aminobutyric acid; Gabra: GABA A receptor subunit; Gat1: GABA transporter 1; Htr: 5-Hydroxytryptamine receptor; MDD: Major depressive disorder; Npy: Neuropeptide Y; PKA: Protein kinase A; Pdyn: Prodynorphin; Pv: Parvalbumin; Trkb: Tropomyosin receptor kinase B; UCMS: Unpredictable chronic mild stress.

## Competing interests

The authors declare that they have no competing interests.

## Authors’ contributions

MS designed the experiment, performed all the mouse-related aspects of the study (surgeries, chronic stress exposure), collected the frontal cortex tissue punches, isolated the RNA, generated the cDNA, performed the qPCR on GABA-related genes, analyzed the data, and wrote the manuscript. KE performed the qPCR on serotonin- and dopamine-related genes, analyzed the associated data, and wrote the manuscript. YD performed the gene network analyses and helped write the manuscript. GT mentored YD on biostatistics analyses. ES helped design the experiment and wrote the manuscript. All authors read and approved the final manuscript.

## Supplementary Material

Additional file 1: Figure S1 Effects of sex-related factors on anxiety-like emotionality *Z*-scores. *Numbers* at the base of *bars* indicate *N*. ***p < 0.001, **p < 0.01; *T* testosterone, *B* blank, *F* gonadal female, *M* gonadal male. Click here for file

Additional file 2: Figure S2 Effects of sex-related factors on expression of GABA-related genes with all eight experimental groups represented separately. *Numbers* at the base of *bars* indicate *N*. ***p < 0.001, **p < 0.01, *p < 0.05, #*p* < 0.1; *T* testosterone, *B* blank, *F* gonadal female, *M* gonadal male. Click here for file

Additional file 3: Figure S3 Effects of sex-related factors on expression of serotonin- and dopamine-related genes with all eight experimental groups represented separately. *Numbers* at the base of *bars* indicate *N*. ***p < 0.001, **p < 0.01, *p < 0.05, #*p* < 0.1; *T* testosterone, *B* blank, *F* gonadal female, *M* gonadal male. Click here for file

Additional file 4: Table S1 Statistical values associated with Pearson correlation analysis of GABA-related genes versus anxiety-like behavior. Numbers in bold indicate comparisons that were significant at 5% false discovery rate with correction for the 14 genes examined. Click here for file

Additional file 5: Table S2 Statistical values associated with Pearson correlation analysis of serotonin/dopamine-related genes versus anxiety-like behavior. Numbers in bold indicate comparisons that were significant at 5% false discovery rate with correction for the 14 genes examined. Click here for file
