# Supplementary figures and images for: Sex chromosome complement regulates expression of mood-related genes
Source: Biol Sex Differ. 2013 Nov 7;4:20. doi: 10.1186/2042-6410-4-20 (PMC4175487; doi:10.1186/2042-6410-4-20)

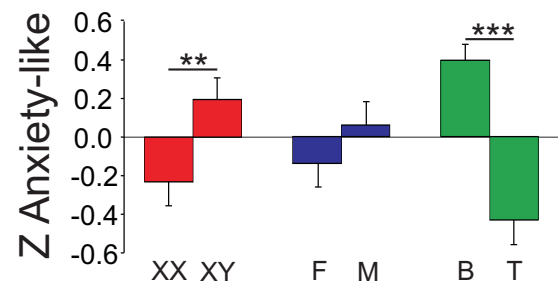

Supplement: Additional file 1: Figure S1 — Effects of sex-related factors on anxiety-like emotionality Z-scores. Numbers at the base of bars indicate N. ***p < 0.001, **p < 0.01; T testosterone, B blank, F gonadal female, M gonadal male. [file 2042-6410-4-20-S1.pdf]

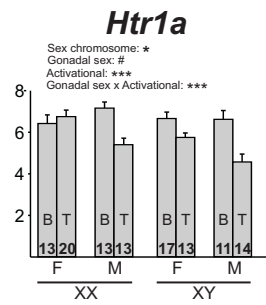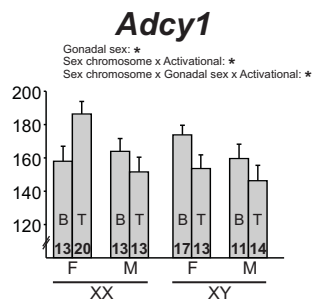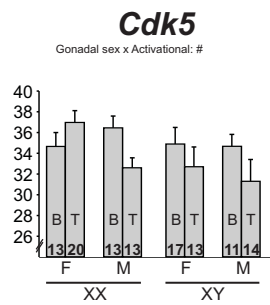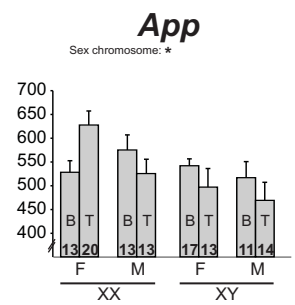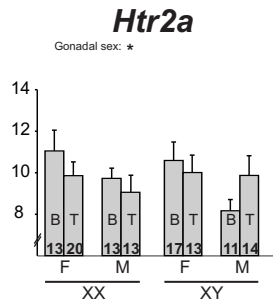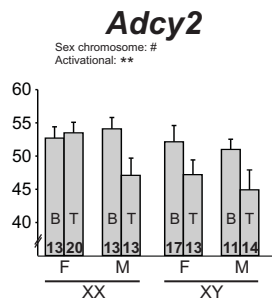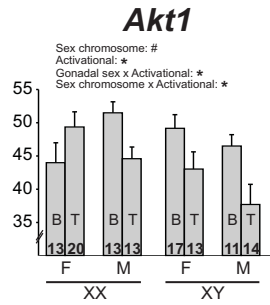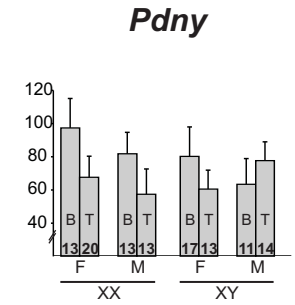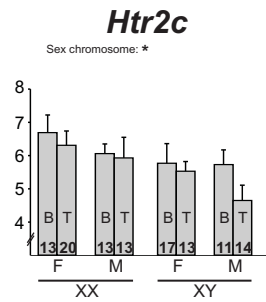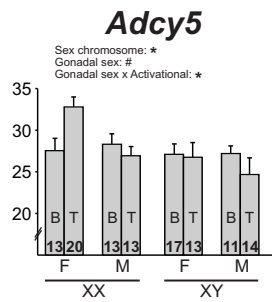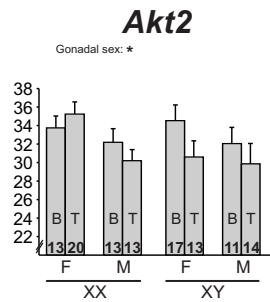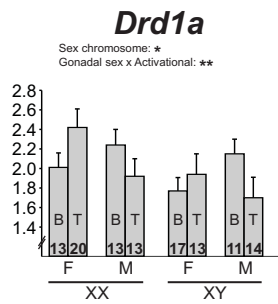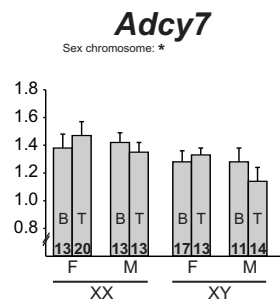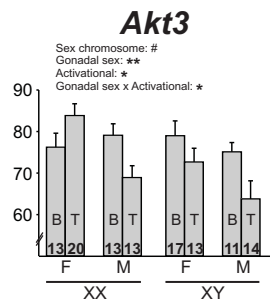

Supplement: Additional file 3: Figure S3 — Effects of sex-related factors on expression of serotonin- and dopamine-related genes with all eight experimental groups represented separately. Numbers at the base of bars indicate N. ***p < 0.001, **p < 0.01, *p < 0.05, #p < 0.1; T testosterone, B blank, F gonadal female, M gonadal male. [file 2042-6410-4-20-S3.pdf]
