# Supplementary material for: Sex chromosome complement regulates expression of mood-related genes
Source: Biol Sex Differ. 2013 Nov 7;4:20. doi: 10.1186/2042-6410-4-20 (PMC4175487; doi:10.1186/2042-6410-4-20)
Supplement: Additional file 2: Figure S2 — Effects of sex-related factors on expression of GABA-related genes with all eight experimental groups represented separately. Numbers at the base of bars indicate N. ***p < 0.001, **p < 0.01, *p < 0.05, #p < 0.1; T testosterone, B blank, F gonadal female, M gonadal male. [file 2042-6410-4-20-S2.pdf]

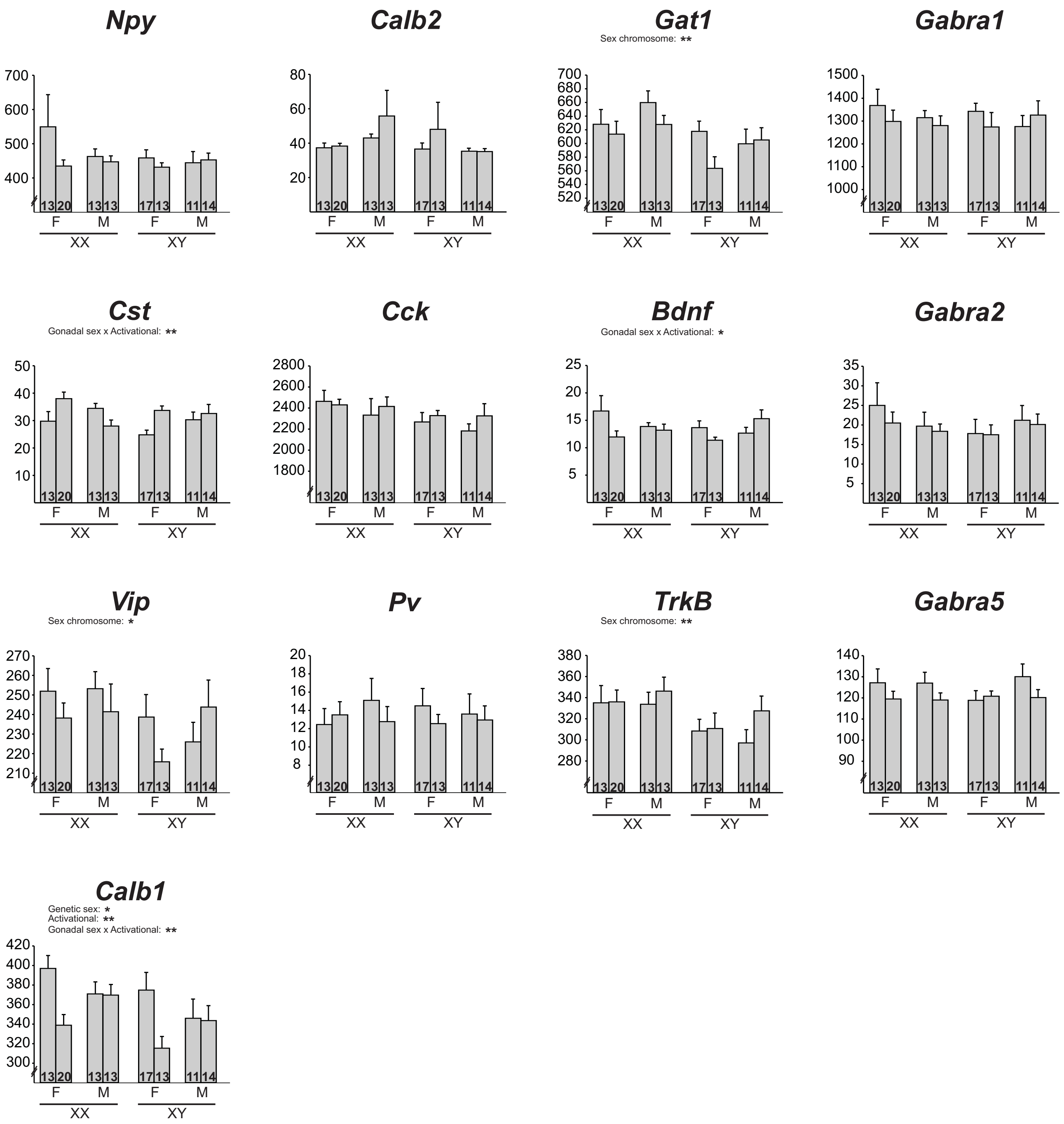
Npy
700
600
500
400
13 20 13 13 17 13 11 14
F M F M
XX XY
Calb2
80
60
40
20
13 20 13 13 17 13 11 14
F M F M
XX XY
Gat1
Sex chromosome: **
700
680
660
640
620
600
580
560
540
520
13 20 13 13 17 13 11 14
F M F M
XX XY
Gabra1
1500
1400
1300
1200
1100
1000
13 20 13 13 17 13 11 14
F M F M
XX XY
Cst
Gonadal sex x Activational: **
50
40
30
20
10
13 20 13 13 17 13 11 14
F M F M
XX XY
Cck
2800
2600
2400
2200
2000
1800
13 20 13 13 17 13 11 14
F M F M
XX XY
Bdnf
Gonadal sex x Activational: *
25
20
15
10
5
13 20 13 13 17 13 11 14
F M F M
XX XY
Gabra2
35
30
25
20
15
10
5
13 20 13 13 17 13 11 14
F M F M
XX XY
Vip
Sex chromosome: *
270
260
250
240
230
220
210
13 20 13 13 17 13 11 14
F M F M
XX XY
Pv
20
18
16
14
12
10
8
13 20 13 13 17 13 11 14
F M F M
XX XY
TrkB
Sex chromosome: **
380
360
340
320
300
280
13 20 13 13 17 13 11 14
F M F M
XX XY
Gabra5
140
130
120
110
100
90
13 20 13 13 17 13 11 14
F M F M
XX XY
Calb1
Genetic sex: *
Activational: **
Gonadal sex x Activational: **
420
400
380
360
340
320
300
13 20 13 13 17 13 11 14
F M F M
XX XY
