# Supplementary material for: Sex chromosome complement regulates expression of mood-related genes
Source: Biol Sex Differ. 2013 Nov 7;4:20. doi: 10.1186/2042-6410-4-20 (PMC4175487; doi:10.1186/2042-6410-4-20)
Supplement: Additional file 4: Table S1 — Statistical values associated with Pearson correlation analysis of GABA-related genes versus anxiety-like behavior. Numbers in bold indicate comparisons that were significant at 5% false discovery rate with correction for the 14 genes examined. [file 2042-6410-4-20-S4.doc]

**Table S1** Statistical values associated with Pearson correlation analysis of GABA-related genes versus anxiety-like behavior. Numbers in bold indicate comparisons that were significant at 5% false discovery rate with correction for the 14 genes examined.

|  | **All mice combined** | **XX** | **XY** | **Gonadal Female** | **Gonadal Male** | **Blank-treated** | **Testosterone-treated** |
| --- | --- | --- | --- | --- | --- | --- | --- |
| ***Sst*** | r=0.12;  p>0.20 | r=0.22;  p<0.1 | r=0.09;  p>0.50 | r=-0.09;  p>0.50 | r=0.32;  p<0.02 | r=0.18;  p>0.20 | r=0.02;  p>0.90 |
| ***Gad67*** | r=-0.003;  p>0.95 | r=-0.003;  p>0.95 | r=0.13;  p>0.30 | r=0.009;  p>0.90 | r=-0.002;  p>0.95 | r=0.03;  p>0.80 | r=-0.05;  p>0.65 |
| ***Gad65*** | r=0.01;  p>0.85 | r=-0.01;  p>0.90 | r=0.23;  p>0.10 | r=0.01;  p>0.90 | r=-0.03;  p>0.80 | r=-0.03;  p>0.80 | r=0.02;  p>0.85 |
| ***Vip*** | r=0.02;  p>0.80 | r=0.09;  p>0.45 | r=0.04;  p>0.75 | r=0.08;  p>0.50 | r=-0.05;  p>0.70 | r=-0.18;  p>0.15 | r=0.07;  p>0.60 |
| ***Calb1*** | r=-0.02;  p>0.80 | r=-0.03;  p>0.80 | r=0.10;  p>0.50 | r=0.16;  p>0.20 | r=-0.25;  p<0.1 | r=-0.27;  p<0.1 | r=-0.11;  p>0.40 |
| ***Cst*** | r=-0.10;  p>0.25 | r=-0.06;  p>0.65 | r=-0.11;  p>0.45 | **r=-0.35;**  **p<0.01** | r=0.20;  p>0.10 | r=0.06;  p>0.65 | r=-0.08;  p>0.55 |
| ***Npy*** | r=0.17;  p<0.10 | r=0.24;  p<0.1 | r=0.07;  p>0.60 | r=0.25;  p<0.1 | r=0.04;  p>0.75 | r=0.20;  p>0.15 | r=0.10;  p>0.45 |
| ***Cr*** | r=-0.11;  p>0.25 | r=-0.26;  p<0.04 | r=0.15;  p>0.30 | r=0.11;  p>0.40 | r=-0.35;  p<0.02 | r=-0.12;  p>0.35 | r=-0.7;  p>0.60 |
| ***Cck*** | r=-0.10;  p>0.30 | r=-0.07;  p>0.55 | r=-0.04;  p>0.75 | r=-0.11;  p>0.40 | r=-0.08;  p>0.55 | r=-0.23;  p<0.1 | r=0.05;  p>0.65 |
| ***Pv*** | r=0.15;  p>0.10 | r=0.23;  p<0.1 | r=0.03;  p>0.80 | r=0.02;  p>0.85 | r=0.26;  p<0.1 | r=0.20;  p>0.15 | r=0.12;  p>0.35 |
| ***Gat1*** | r=-0.08;  p>0.40 | r=-0.05;  p>0.70 | r=0.02;  p>0.85 | r=-0.13;  p>0.30 | r=-0.06;  p>0.65 | r=-0.20;  p>0.10 | r=-0.16;  p>0.20 |
| ***Gabra1*** | r=0.03;  p>0.75 | r=0.22;  p<0.1 | r=-0.25;  p<0.1 | r=0.09;  p>0.50 | r=-0.05;  p>0.70 | r=0.06;  p>0.60 | r=-0.04;  p>0.75 |
| ***Gabra2*** | r=-0.04;  p>0.65 | r=0.04;  p>0.75 | r=-0.16;  p>0.25 | r=-0.04;  p>0.75 | r=-0.04;  p>0.75 | r=0.03;  p>0.85 | r=-0.18;  p>0.15 |
| ***Gabra5*** | r=0.15;  p>0.10 | r=0.22;  p<0.1 | r=0.04;  p>0.75 | r=-0.03;  p>0.80 | r=0.26;  p<0.1 | r=-0.02;  p>0.95 | r=0.17;  p>0.20 |
| ***Bdnf*** | r=0.08;  p>0.35 | r=0.07;  p>0.55 | r=0.13;  p>0.30 | r=0.13;  p>0.30 | r=0.003;  p>0.95 | r=-0.18;  p>0.20 | r=0.18;  p>0.15 |
| ***Trkb*** | **r=-0.28; p<0.003** | r=-0.32;  p<0.02 | r=-0.11;  p>0.40 | r=-0.28;  p<0.04 | r=-0.30;  p<0.03 | r=-0.25;  p<0.1 | r=-0.28;  p<0.03 |
