# Supplementary material for: Sex chromosome complement regulates expression of mood-related genes
Source: Biol Sex Differ. 2013 Nov 7;4:20. doi: 10.1186/2042-6410-4-20 (PMC4175487; doi:10.1186/2042-6410-4-20)
Supplement: Additional file 5: Table S2 — Statistical values associated with Pearson correlation analysis of serotonin/dopamine-related genes versus anxiety-like behavior. Numbers in bold indicate comparisons that were significant at 5% false discovery rate with correction for the 14 genes examined. [file 2042-6410-4-20-S5.doc]

**Table S2** Statistical values associated with Pearson correlation analysis of serotonin/dopamine-related genes versus anxiety-like behavior. Numbers in bold indicate comparisons that were significant at 5% false discovery rate with correction for the 14 genes examined.

|  | **All mice combined** | **XX** | **XY** | **Gonadal Female** | **Gonadal Male** | **Blank-treated** | **Testosterone-treated** |
| --- | --- | --- | --- | --- | --- | --- | --- |
| ***Htr1a*** | r=0.04;  p>0.65 | r=-0.04;  p>0.75 | **r=0.30;**  **p<0.04** | r=-0.16;  p>0.20 | **r=0.26;**  **p<0.1** | r=-0.17;  p>0.20 | r=-0.13;  p>0.35 |
| ***Htr2a*** | r=0.06;  p>0.50 | r=0.15;  p>0.25 | r=-0.05;  p>0.75 | r=0.13;  p>0.30 | r=0.02;  p>0.85 | r=-0.09;  p>0.50 | r=0.12;  p>0.35 |
| ***Htr2c*** | **r=-0.22;**  **p<0.03** | **r=-0.35;**  **p<0.01** | r=0.12;  p>0.40 | **r=-0.27;**  **p<0.04** | r=-0.13;  p>0.35 | r=-0.19;  p>0.15 | **r=-0.36;**  **p<0.01** |
| ***Drd1a*** | r=-0.13;  p>0.15 | r=-0.17;  p>0.15 | r=0.04;  p>0.75 | **r=-0.38; p<0.005** | r=0.16;  p>0.20 | r=-0.03;  p>0.80 | r=-0.21;  p>0.10 |
| ***Adcy1*** | r=-0.03;  p>0.75 | r=-0.13;  p>0.30 | r=0.23;  p>0.1 | **r=-0.26;**  **p<0.05** | **r=0.29;**  **p<0.04** | r=0.04;  p>0.75 | r=-0.08;  p>0.50 |
| ***Adcy2*** | r=0.02;  p>0.80 | r=-0.07;  p>0.55 | **r=0.24;**  **p<0.1** | **r=-0.25;**  **p<0.1** | **r=0.31;**  **p<0.03** | r=-0.21;  p>0.1 | r=-0.05;  p>0.65 |
| ***Adcy5*** | **r=-0.17;**  **p<0.1** | **r=-0.37; p<0.005** | r=0.24;  p<0.1 | **r=-0.42; p<0.001** | r=0.17;  p>0.20 | r=-0.14;  p>0.30 | r=-0.17;  p>0.15 |
| ***Adcy7*** | r=-0.14;  p>0.10 | r=-0.17;  p>0.15 | r=0.05;  p>0.70 | **r=-0.36;**  **p<0.01** | r=0.14;  p>0.30 | r=-0.15;  p>0.25 | r=-0.17;  p>0.15 |
| ***Cdk5*** | r=0.08;  p>0.40 | r=-0.08;  p>0.50 | **r=0.37;**  **p<0.01** | r=-0.11;  p>0.35 | **r=0.32;**  **p<0.02** | r=-0.05;  p>0.70 | r=0.05;  p>0.70 |
| ***Akt1*** | r=0.01;  p>0.90 | r=-0.18;  p>0.10 | **r=0.40;**  **p<0.005** | **r=-0.25;**  **p<0.1** | **r=0.32;**  **p<0.02** | r=-0.008;  p>0.95 | r=-0.13;  p>0.30 |
| ***Akt2*** | r=0.008;  p>0.90 | r=-0.14;  p>0.25 | **r=0.26;**  **p<0.1** | r=-0.10;  p>0.40 | r=0.18;  p>0.15 | r=-0.07;  p>0.60 | r=-0.03;  p>0.80 |
| ***Akt3*** | r=-0.05;  p>0.60 | r=-0.18;  p>0.15 | **r=0.25;**  **p<0.1** | **r=-0.31;**  **p<0.02** | **r=0.30;**  **p<0.03** | r=-0.10;  p>0.45 | r=-0.14;  p>0.25 |
| ***App*** | **r=-0.18;**  **p<0.1** | **r=-0.30;**  **p<0.02** | r=0.13;  p>0.35 | **r=-0.42; p<0.001** | r=0.09;  p>0.50 | r=-0.09;  p>0.50 | **r=-0.26;**  **p<0.05** |
| ***Pdyn*** | r=0.13;  p>0.15 | **r=0.31;**  **p<0.02** | r=-0.14;  p>0.30 | r=0.17;  p>0.15 | r=0.10;  p>0.45 | r=-0.02;  p>0.90 | r=0.15;  p>0.25 |
